# Supplementary figures and images for: Live imaging of the Cryptosporidium parvum life cycle reveals direct development of male and female gametes from type I meronts
Source: PLoS Biol. 2022 Apr 18;20(4):e3001604. doi: 10.1371/journal.pbio.3001604 (PMC9015140; doi:10.1371/journal.pbio.3001604)

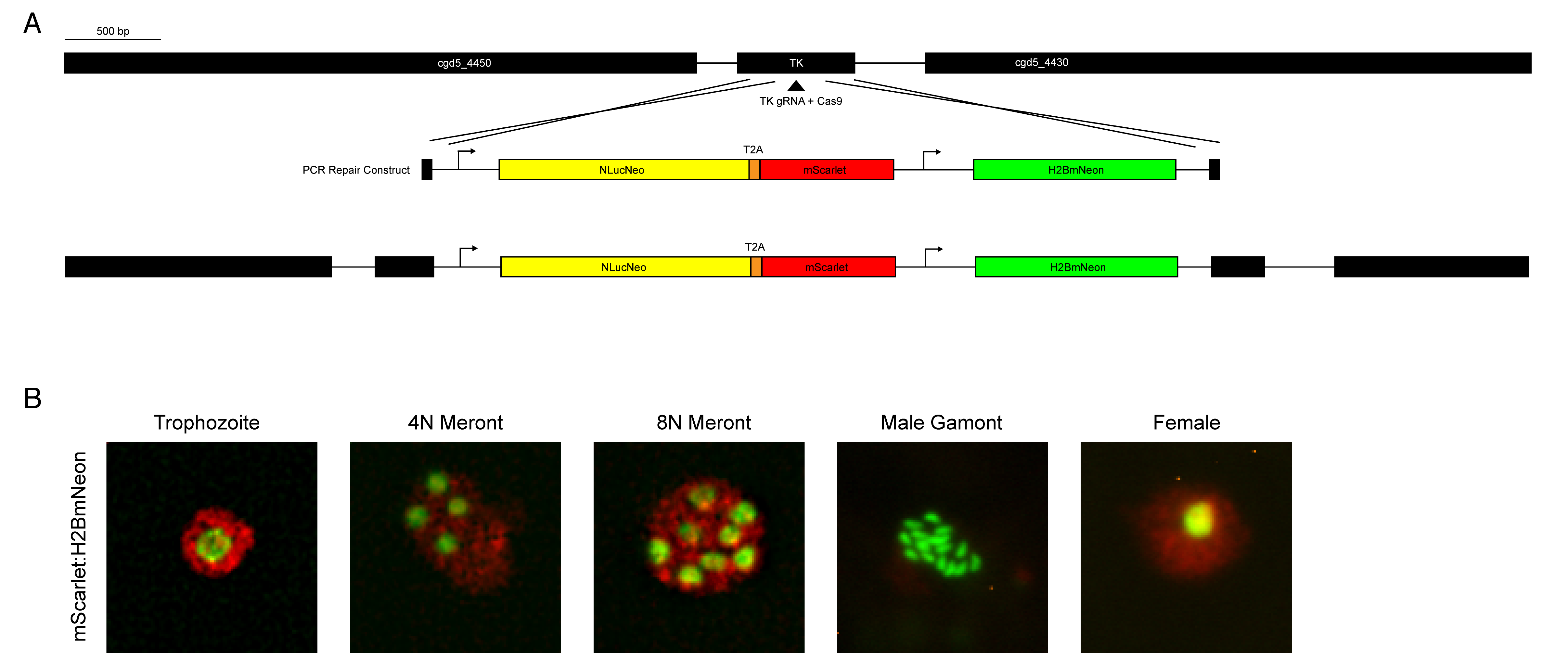

Supplement: S1 Fig — (A) Schematic overview of the guide and repair constructs used to generate the transgenic parasite line with a cytosolic mScarlet and a nuclear mNeon inserted into the TK locus. (B) Visualization of the fluorescent protein localization in multiple life stages for the mScarlet-H2BmNeon transgenic parasites. TK, thymidine kinase. (TIF) [file pbio.3001604.s001.tif]

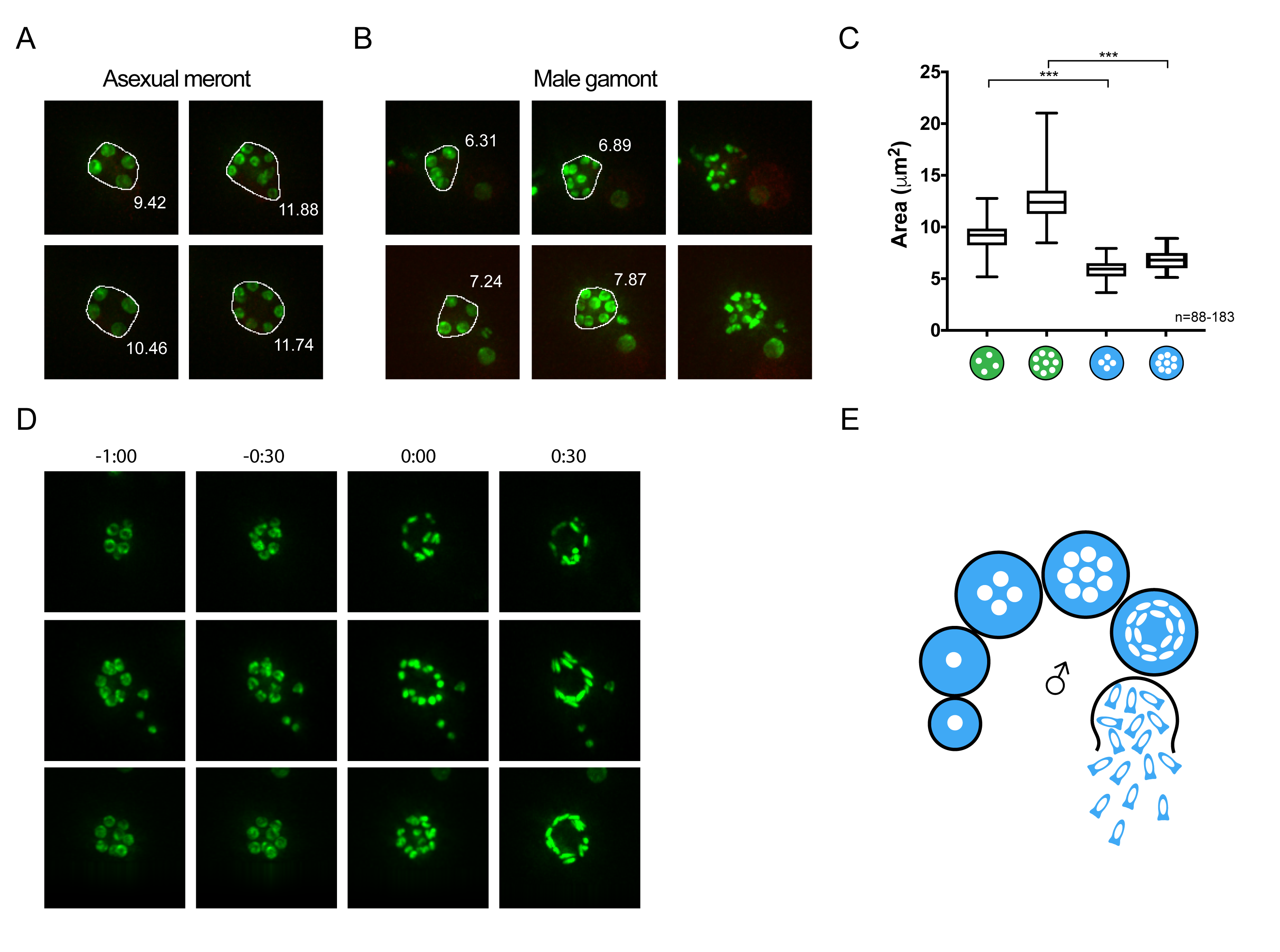

Supplement: S2 Fig — (A) Two representative images depicting asexual parasites at 4 and 8 nuclei, including the boundary drawn around all nuclei to measure nuclear spread. The area for each meront is included. (B) Representative images depicting male parasites at 4, 8, and then 16 nuclei, including the boundary drawn around all nuclei at 4 and 8 nuclei stages to measure nuclear spread. The area for each gamont is included. (C) Comparison of the area of nuclear spread for asexual and male parasites with 4 or 8 nuclei. The area taken up by male nuclei is significantly smaller than the area taken up by asexual nuclei at both the 4 and 8 nuclei stages (Welch t test, *** p< 0.0001). (D) Three representative image series of male nuclear development. Nuclei remain round when 8 nuclei are present and adopt distinct bullet-like male shape only after dividing to 16 nuclei. (E) Schematic representation of the development of the male gamont. Underlying data are provided in the Supporting information as S5 Data. (TIF) [file pbio.3001604.s002.tif]

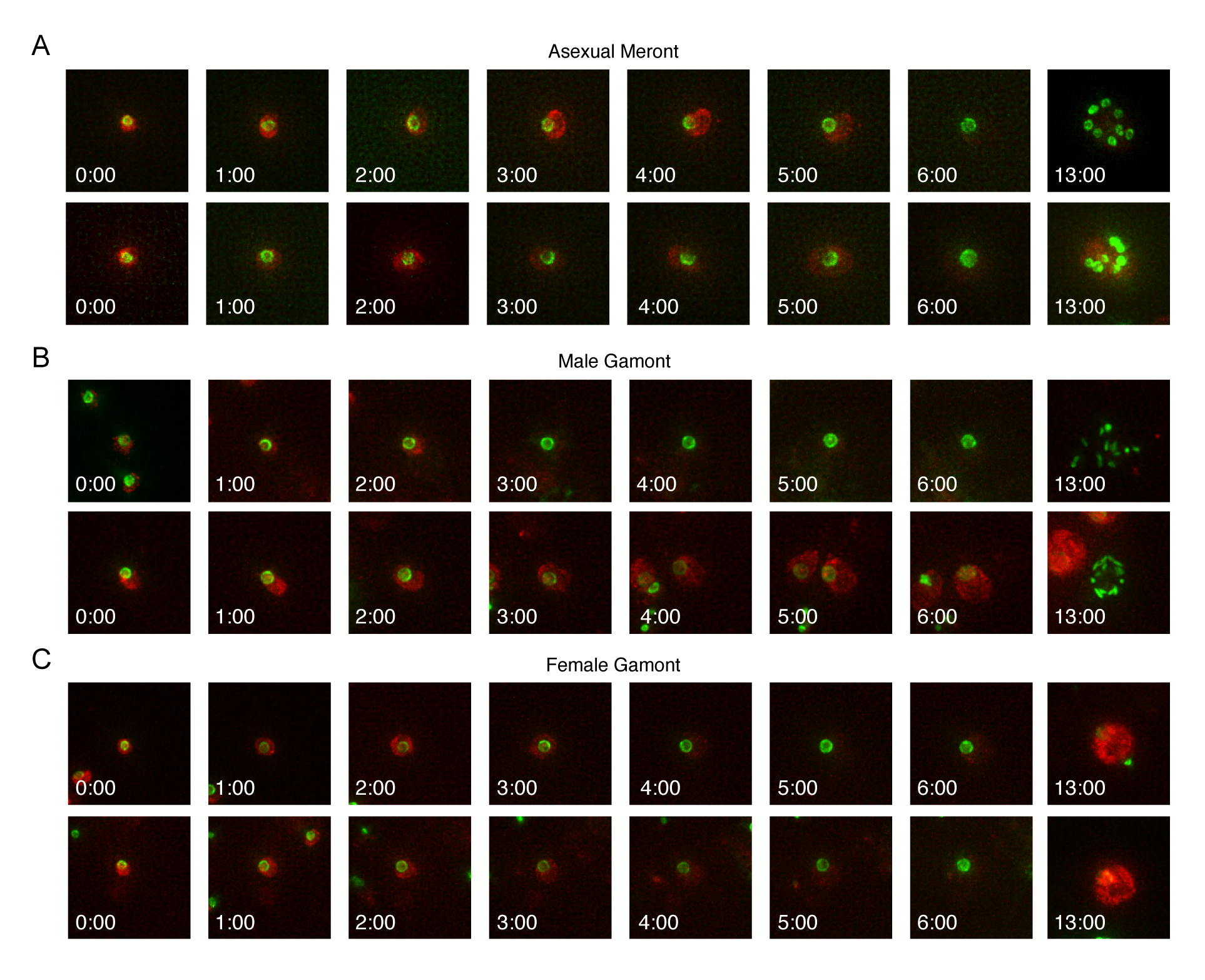

Supplement: S3 Fig — (A) Images from 2 representative asexual meronts shown every hour for the first 6 hours, followed by a 13-hour time point to confirm stage. (B) Images from 2 representative male gamonts shown every hour for the first 6 hours, followed by a 13-hour time point to confirm stage. (C) Images from 2 representative female gamonts shown every hour for the first 6 hours, followed by a 13-hour time point to confirm stage. (TIF) [file pbio.3001604.s003.tif]
