# Supplementary material for: Live imaging of the Cryptosporidium parvum life cycle reveals direct development of male and female gametes from type I meronts
Source: PLoS Biol. 2022 Apr 18;20(4):e3001604. doi: 10.1371/journal.pbio.3001604 (PMC9015140; doi:10.1371/journal.pbio.3001604)
Supplement: Attachment — Submitted filename: Rebuttal_English.docx [file pbio.3001604.s013.docx]

We thank all three reviewers for their enthusiasm for the importance of the findings and their very kind works on the overall impact and quality of the work. The reviewers also made helpful suggestions to enhance the clarity of the writing and some figure elements. We essentially followed all these suggestions and respond to each of them point-by-point below.

Reviewer #1:

The only part of the paper that needs revision is the discussion, which, in stark contrast to the rest of the paper, is not well written. There are many run on sentences, sentences that are grammatically incorrect or simply unclear. "This" is used frequently without a noun following, which adds to the lack of clarity. There are missing commas, singular instead of plural words used (oocyst instead of oocysts) Additionally the discussion runs for 5.5 pages; it should be condensed considerably.

*We revised the discussion section to improve its clarity and grammar.*

Reviewer #2:

Some suggestions:

1)	The language in the abstract "We determine that the parasite executes an intrinsic program of three generations of asexual replication, followed by a single generation of sexual stages" is somewhat stronger than the "we propose that". The data for this experimental setup are very strong, but they don't exclude the possibility of other programs in other environmental conditions (including other in vivo scenarios), and I would encourage the use of some qualification of this statement in the abstract. Does this apply to all species? Is it true in other culture systems?

*We tempered the strength of the conclusion as suggested by the reviewer by using ‘we propose’ instead of ‘we determine’.*

2)	Figure 1

I didn't find 1A very clear - presumably this is meant to convey the two possibilities that commitment to sexual stages could be either through an external induction or intrinsic or time based pathway. But the arrows confused me - I initially processed this as a vertical arrow (pathway) coming down from "Extrinsic or intrinsic lifecycle progression" to the first green circle/parasite, and then found the other arrows confusing. Perhaps the source of confusion is that identically styled arrows in this diagram indicate transition from one parasite life stage to another as well as indicating external stimuli. Maybe it could be made clearer that there are two hypothesised pathways to commitment, with alternate arrows running through each pathway, while the external stimuli are symbolised using another symbol (lightning bolt? Or a different type of arrow)

*We modified this panel to more clearly convey the concept of two alternative hypotheses by placing them side by side.*

3)	I found the discussion starting at line 405 for the origin of the type II meront useful and interesting. However, I would appreciate a bit more detail here - the inference in this discussion is that the concept is an error based only on inference from the related Eimeria, but is there any other evidence for or against the existence of type II meronts? Is there other literature that tried to find these, or just repeated re-citation of the original Vetterling speculation that became inappropriately more dogmatic with time?

*We ourselves, in the process of writing, had the same question. There is surprisingly little direct evidence for the type 2 meront. Studies following the Vetterling papers often show a parasite with four nuclei and declare it a type 2 meront. There is little to no exploration of the possibility that these may be either intracellular oocysts or immature type 1 meronts or male gamonts. We believe it likely that investigators mistook immature male stages as type 2 meronts as these are observable just before mature gamonts and differ morphologically from meronts in that their nuclei form a central cluster and they are. However, we are hesitant to put words or interpretations in the mouths of previous authors. We feel our statement here is balanced: Tyzzer reports 8N, Vetterlig 4N … this is difficult to resolve using fixed samples … therefore we image living cells and our data is consistent with Tyzzer’s model.*

Minor Issues

4)	I am not a Latin scholar, but the possessive s added to the end of a species or genus name (e.g. "Cryptosporidium's" at line 25 seems wrong to me. I would reword this to avoid that usage. I don't think a possessive S can be added to a formal scientific name, and several (admittedly unauthoritative ) style guides agree e.g. https://urldefense.com/v3/__https://animaldiversity.org/teach/editor_guidelines/__;!!IBzWLUs!EjsxuUCHRqPLSkwDGm_9E2uPqQl_gQxwy8Llis20_-iNJ3QY_hkTxa8Ze_ihqK6p$

https://urldefense.com/v3/__https://markscherz.tumblr.com/post/85644377348/plurals-and-possessives-in-taxonomic-nomenclature__;!!IBzWLUs!EjsxuUCHRqPLSkwDGm_9E2uPqQl_gQxwy8Llis20_-iNJ3QY_hkTxa8Ze5um33U6$

*Boris ‘enjoyed’ 5 years of Latin in high school, 40 years later, here is the little he remembers: There is no possessive ‘s in Latin, but there is declension that results in different endings of nouns. Assuming that Cryptosporidium follows gender-neutral o-declension the ending would be Cryptosporidi to indicate possession by use of the genitive. Boris fully agrees with the reviewer … best to find another way to say this … ‘the entire Cryptosporidium lifecycle unfolds’ …*

5)	Figure 3

Although clearly described in the figure legend, some titles (beyond individual letters) above each panel would be helpful to avoid having to go back and forth between legend and graphic - e.g. "asexual" above panel B, "males" above C?

*We added annotation as suggested.*

6)	I don't think the usage of "sexualises" in line 78 or "sexualized" at line 154 is correct. These normally mean to attribute a sexual role, but in these cases the meaning is that the parasites have been committed to a sexual lineage (or includes some parasites that are committed). I recommend rewording.

*We reworded the statement.*

7)	The sentence at line 97 reads strangely "The Cryptosporidium lifecycle is remarkably short and much of it unfolds in tissue culture." This gives the partial sense that the lab tissue flask is the natural habitat of the parasite. Do you mean that much of the lifecycle can be recapitulated in vitro/ ex vivo?

*Yes, we meant to indicate that, except for fertilization and oocyst formation, the entire lifecycle can be observed in culture. We reworded to make this clearer.*

8)	Line 183 "decerned". Decern is either archaic, or more recently has a judicial meaning that is not intended here. Discern is the relevant homophone.

*Corrected.*

9)	I didn't understand the bit at lines 308-310

"Based on our observations, the 95% confidence interval suggested that out of the 8 progeny in each meront, between 1.92 and 3.11 will be male. This is consistent with the fact that we never observed more than 2 male progeny from a single meront." If the confidence interval suggest that 3 of the 8 meronts can be male, why is this consistent with never seeing more than 2 males?

*Because we usually only observe half of the progeny. We agree this is overly complicated. We now just state what the confidence interval is.*

10)	Line 139 "We also conducted experiments using a C. parvum reporter strain [21]"

I think this needs a bit more explanation without the reader having to go to this paper - e.g. that the strain expresses markers tagging male (Hap2?) and female (cowp1?) specific genes that allows the observer to discern male vs female gametes.

*This is explained better now*.

11) line 464 contrast should be contrasts

*Corrected.*

12) line 500 - see Nat Microbiol . 2019 Jan;4(1):144-154 for a potential exception (for mixed asexual and sexual, which would infer potential for later mix of male and female, though not directly from the same parasite as seen in this study

*We have added this reference suggested by the reviewer.*

13) Figure 5 - Perhaps the figure would benefit from some arrows to more explicitly depict the sequence, as well as some annotation to clarify that the oocyst is a source of ongoing autoinfection as well as an infectious form that exits the host - I think this could be important for the many readers who aren't already familiar with the lifecycle.

a

*We assume the reviewer meant Figure 6 here. We added arrows to indicate direction.*

Reviewer #3 (Guan Zhu):

I have no major concerns on the data and conclusions, but a few minor comments as described below.

1) Figure 1, E-F (Super-infection assay data): Infection at 0 h = infection with H2bNeon strain of C. parvum (Cp) only; infection at 24 h = Mixed infections with both WT Cp (0 h) and H2bNeon strain Cp (infection at 24 h post-infection with WT Cp. It may be better to have a timeline bar (or may be another way) to clarify the two different types of h/hours in the figures.

*We revised these panels and their annotation to make this clearer.*

2) Line 178 "found a mean time to egress of 12.57 hours": from which time point (e.g., from the recording of images at 11 hpi after inoculation of sporozoites, total = 11 + 12.57 = 23.57 hpi)?

*This is the duration of intracellular development from invasion (first appearance of a new intracellular stage) to egress (disappearance of the intracellular stage). We make this clearer now in the description.*

3) Figure 2 (time points in lapse images and bar charts): In each panel, please clarify/calibrate the start point of the time in relationship to the h post-infection (hpi). Based on the description in lines 167-169 "We began imaging at 11 hours of cultures … also … imaging from 29 or 40 hours of culture onwards", the 0 h or 0:00 h start point could be equivalent to 11, 29 or 40 hpi.

*We clarified this point.*

4) Lines 208-218 (microgamete development): Please clarify the number of observed cell cycles for male parasites (4 cycles of cell division?).

*The reviewer might have overlooked this in line 210: “The development of male gamonts followed a pattern similar to that of meronts (Fig 2B and S2 Movie). After a 6.28-hour establishment phase, four rapid mitotic cycles produced 16 nuclei, and the average time to egress was 12.08 h (Fig 2F, G and H).*

5) Figure 3A, Pink/red lines: Pink lines are not well shown. Will another color work better, e.g., orange, plus some use of opacity?

*Could this be an issue with the low-resolution version of the figure in the pdf file? In the original this is highly visible. Just to clarify there is no opacity set here as the lines do not overlap.*

6) Figure 4: Please clarify the time points in each panel (equal to hpi or equal to +11 hpi). Figure 4B: Please clarify the meaning of the daggers. Figure 4C: Please clarify the "grey" zones (non-egressed merozoites?). Do all tracked meronts produced 8 merozoites?

*All meronts had 8 nuclei prior to egress. We do not directly observe egress in each case, so we don’t have a formal count of merozoites. We do not unambiguously track all progeny in the next generation (and we discuss this in detail). The grey bar indicates the number of nuclei in each meront for which we are unable to observe or unambiguously assign a fate in the next generation. The crosses symbolize cells that die during observation. We make this clearer now.*

7) Please define the "hours" in the manuscript when necessary (relationship with h post-infection [hpi]). Also most the words "hours" may be changed to "h".

*We did this throughout the manuscript as suggested.*

8) Figure 6A: It may help to add arrows to between developmental stages, and mark both male and female gamonts and gametes. In current form, it appears that the female gametes are developed from male gamonts.

*We added arrows to enhance clarity.*
